# Supplementary figures and images for: GUESS-ing Polygenic Associations with Multiple Phenotypes Using a GPU-Based Evolutionary Stochastic Search Algorithm
Source: PLoS Genet. 2013 Aug 8;9(8):e1003657. doi: 10.1371/journal.pgen.1003657 (PMC3738451; doi:10.1371/journal.pgen.1003657)

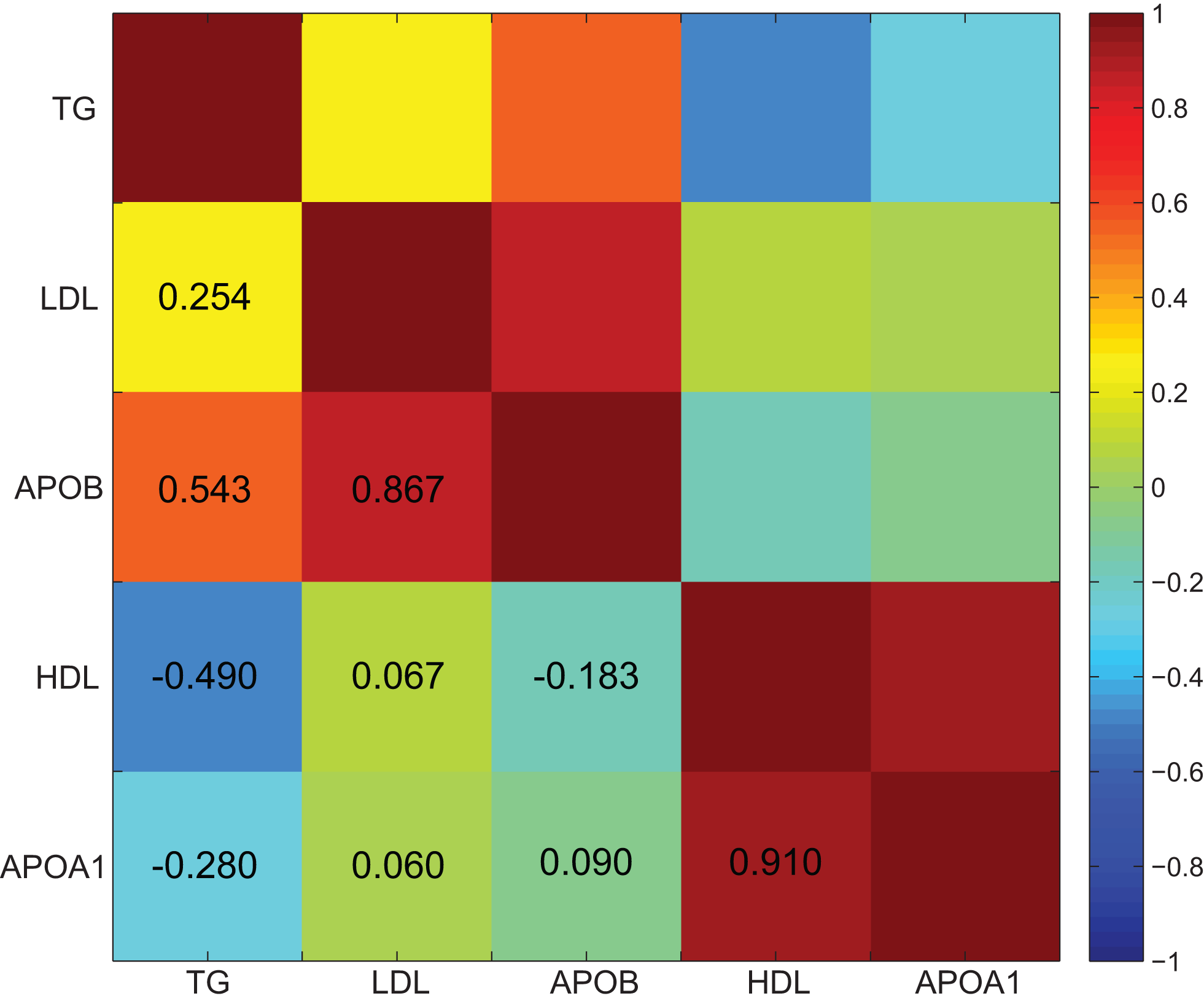

Supplement: Figure S1 — Heat-map of the correlation matrix of the five traits used in the tree analysis. Off-diagonal correlation between each pair of traits is indicated inside the heat-map. (TIF) [file pgen.1003657.s001.tif]

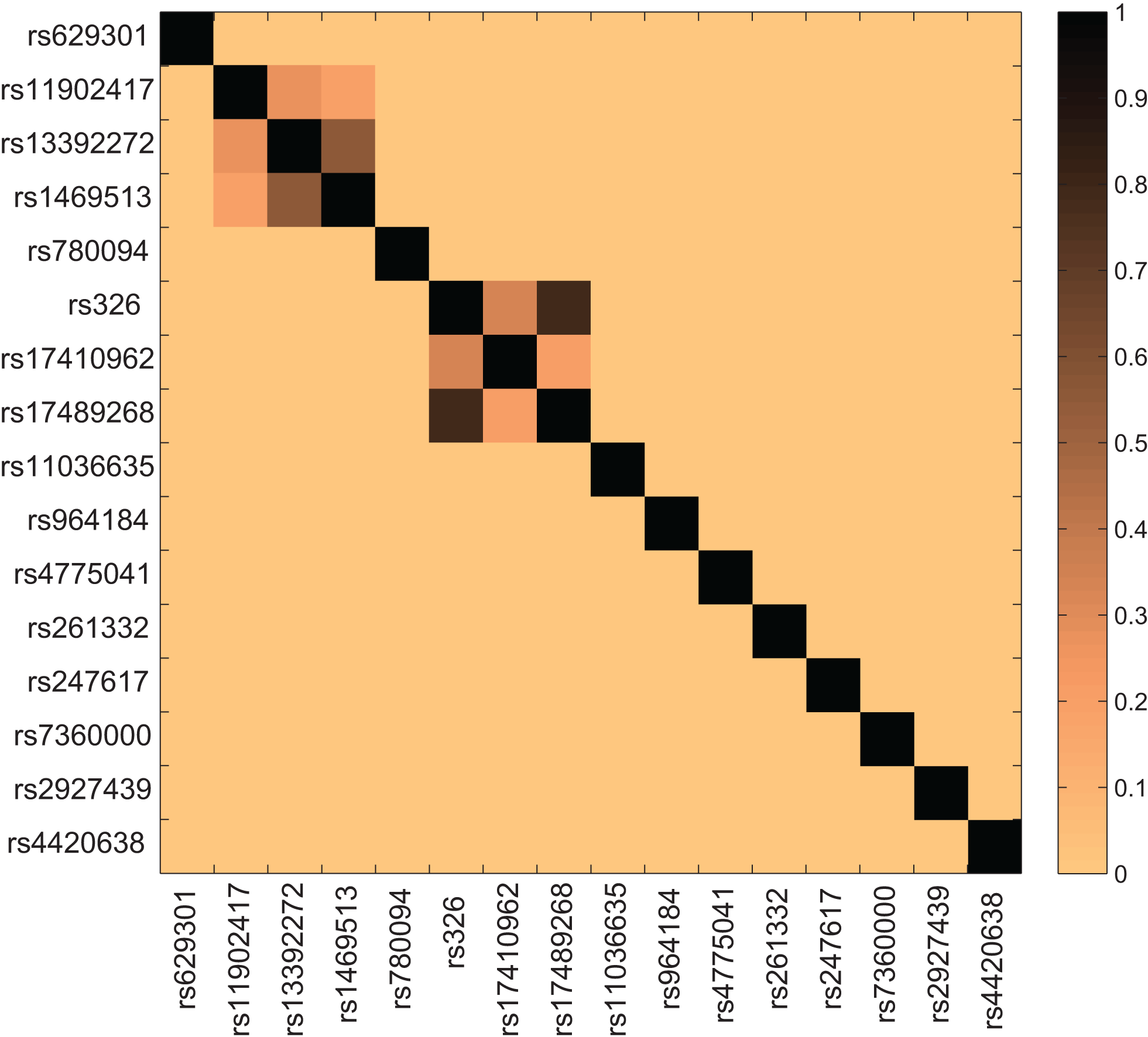

Supplement: Figure S2 — Heat-map of the squared correlation matrix of the 16 SNPs which were marginally associated with any group of traits using an empirical FDR cut-off of 5%. Squared correlation between rs11902417 and rs13392272 is 0.2711, rs11902417 and rs1469513 is 0.1949 and rs13392272 and rs1469513 is 0.5582. Squared correlation between rs326 and rs17410962 is 0.3305, rs326 and rs17489268 is 0.7901 and rs17410962 and 17489268 is 0.2165. (TIF) [file pgen.1003657.s002.tif]

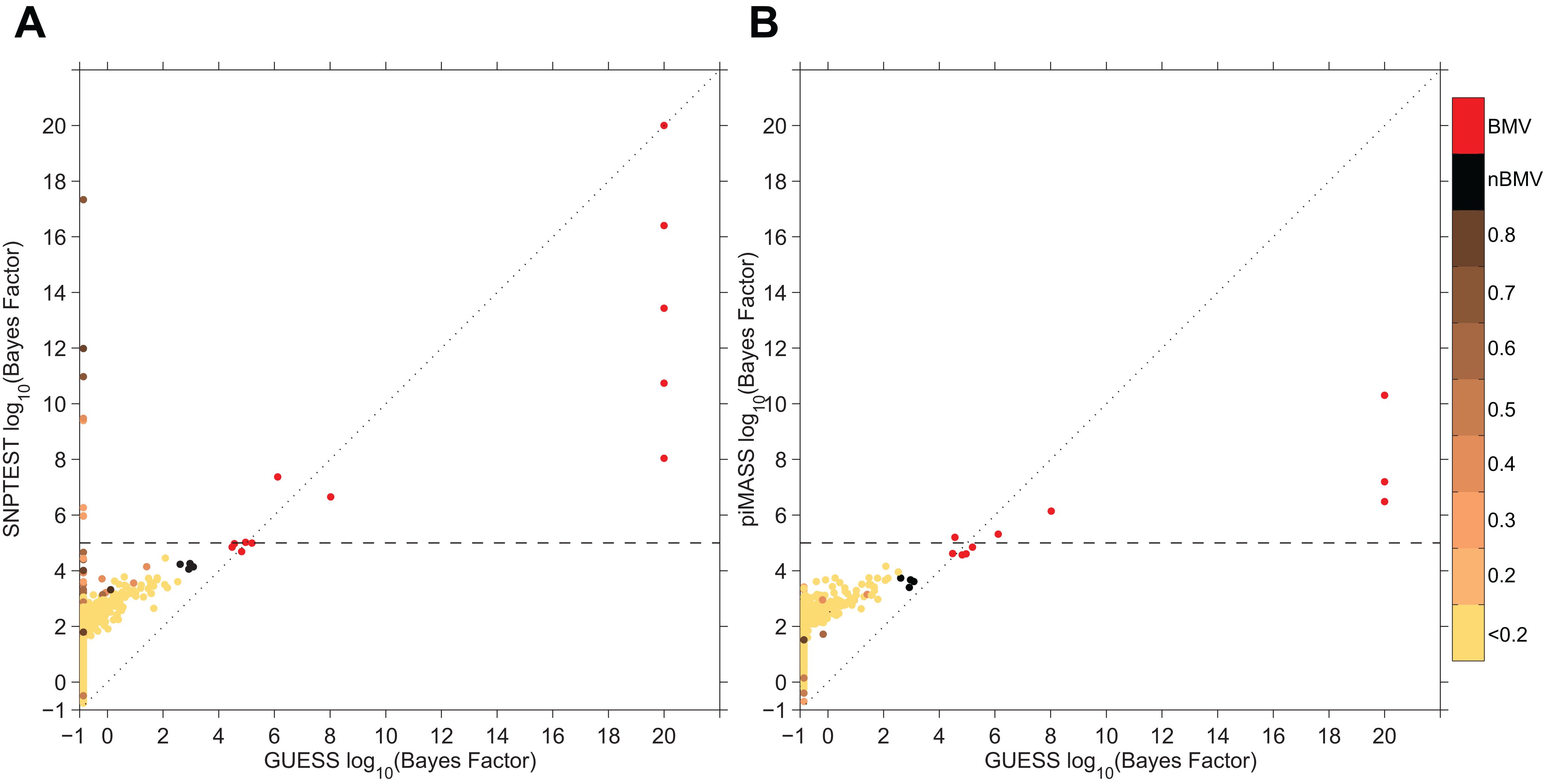

Supplement: Figure S3 — Comparison of the marginal phenotype-SNP association provided by GUESS, SNPTEST and piMASS for all single traits of two trees. (To increase readability, the log10(BFs) are truncated at 20). (A) Scatterplot of log10(BF) GUESS vs SNPTEST obtained superimposing the scatterplot of each single trait. A horizontal dashed line indicates level of log10(BF) that provides strong evidence of a phenotype-SNP association (log10(BF)>5). Red and black dots highlight significant SNPs found by GUESS while non-significant SNPs are colour coded according to the level of pairwise Pearson correlation with the closest significant GUESS SNP (see the colour bar for the correlation scale). (B) Scatterplot of log10(BF) GUESS vs piMASS obtained superimposing the scatterplot of each single trait. Colour code used to identify relevant SNPs and horizontal dashed line are the same as defined in (A). (TIF) [file pgen.1003657.s003.tif]

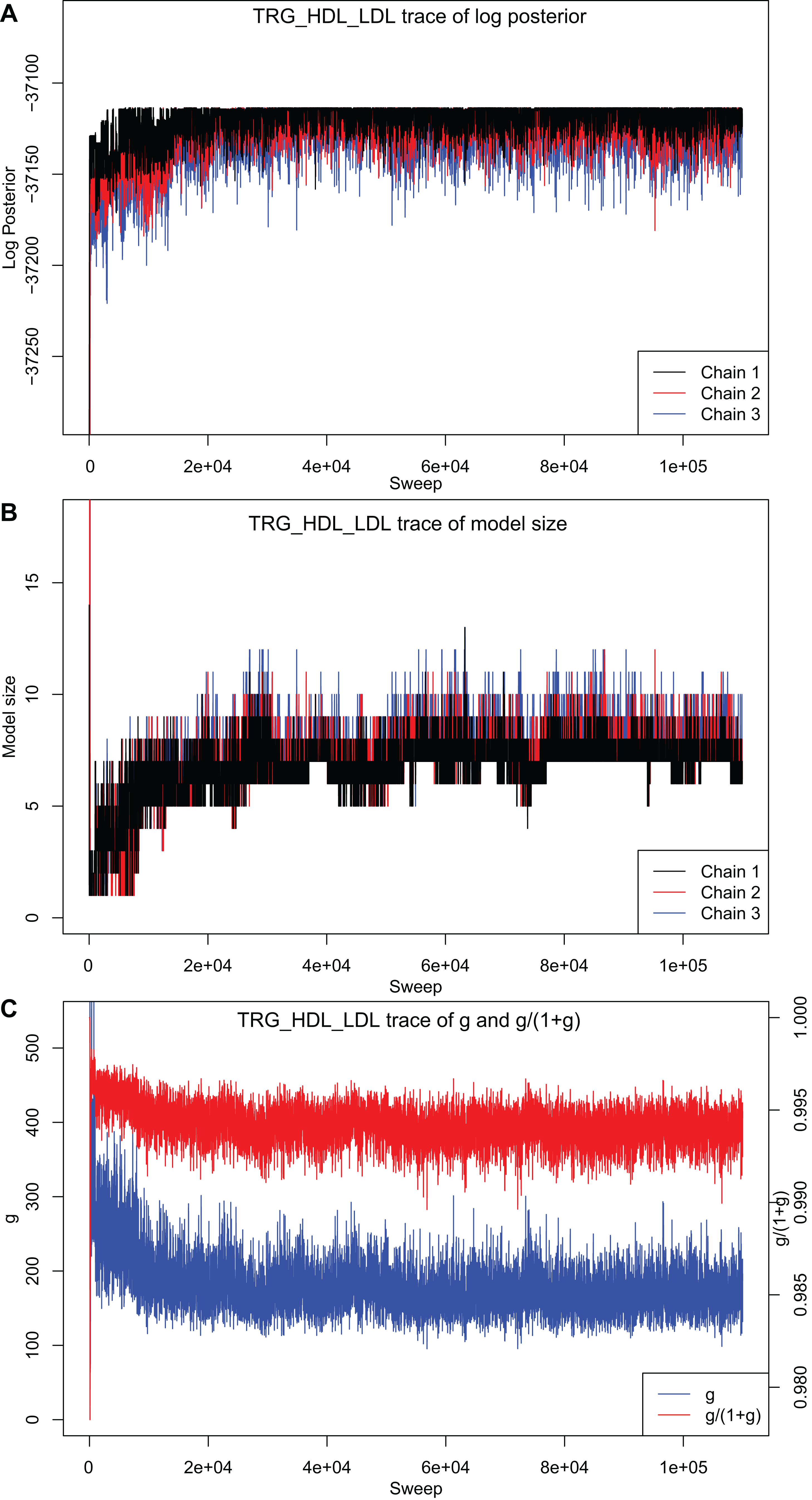

Supplement: Figure S4 — GUESS diagnostic plots in the TG-HDL-LDL group analysis. (A) Trace plot of the log-Posterior (log-marginal likelihood×log-prior on the model space) of the three chains run in parallel. (B) Trace plot of the size of the models explored by the three chains run in parallel. (C) Trace plot of the selection coefficient g (blue) and shrinkage factor g/(1+g). In all plots, black vertical dotted line indicates the end of the burn-in phase. (TIF) [file pgen.1003657.s004.tif]

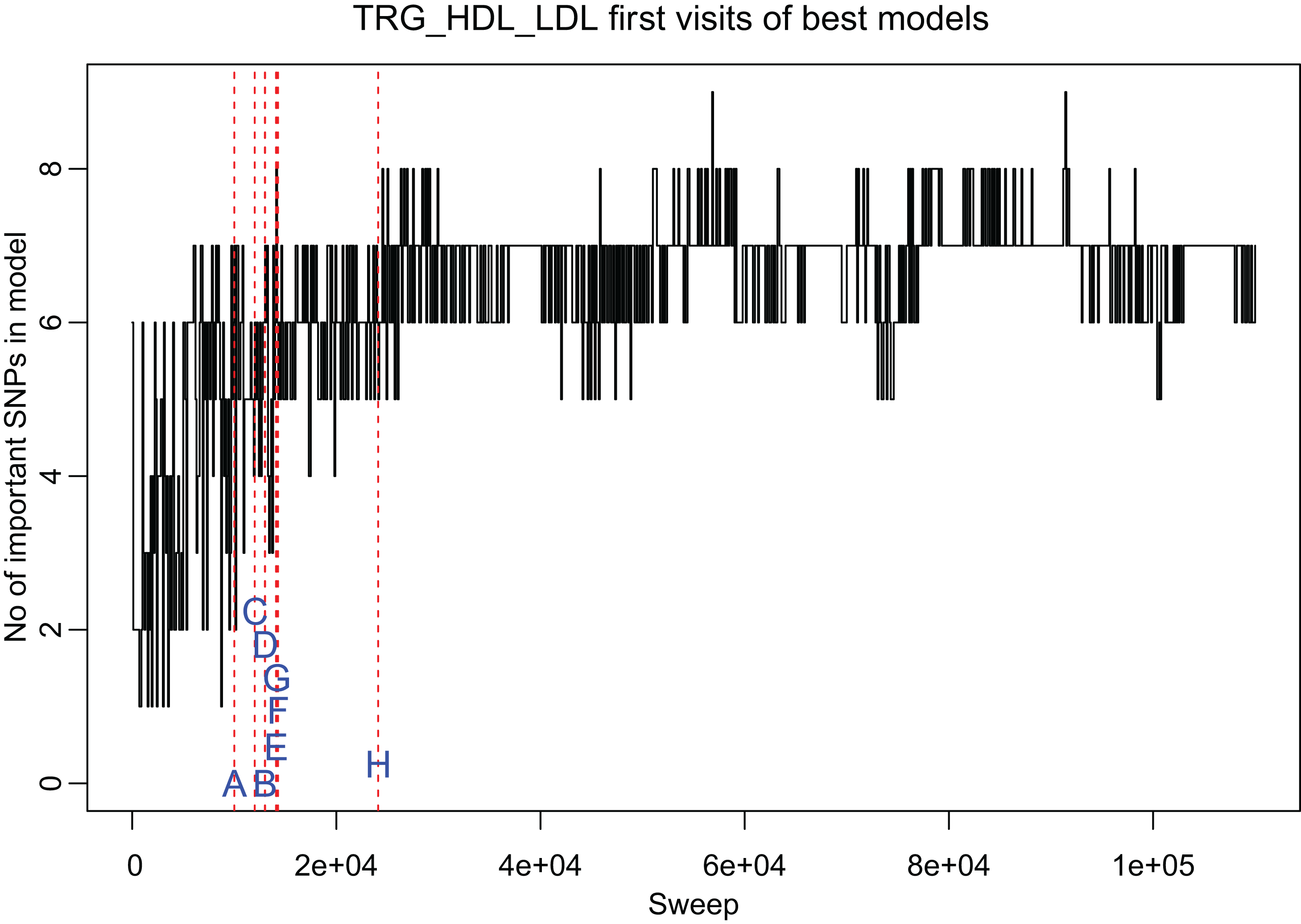

Supplement: Figure S5 — Trace plot of the size of the models explored by the non-heated chain of GUESS in the TG-HDL-LDL group analysis. Letters A-H indicate when GUESS first identifies the top Best Model Visited (A), the second Best Model Visited (B) and etc. with models ranked by the Model Posterior Probability. A black vertical dotted line indicates the end of the burn-in phase. (TIF) [file pgen.1003657.s005.tif]

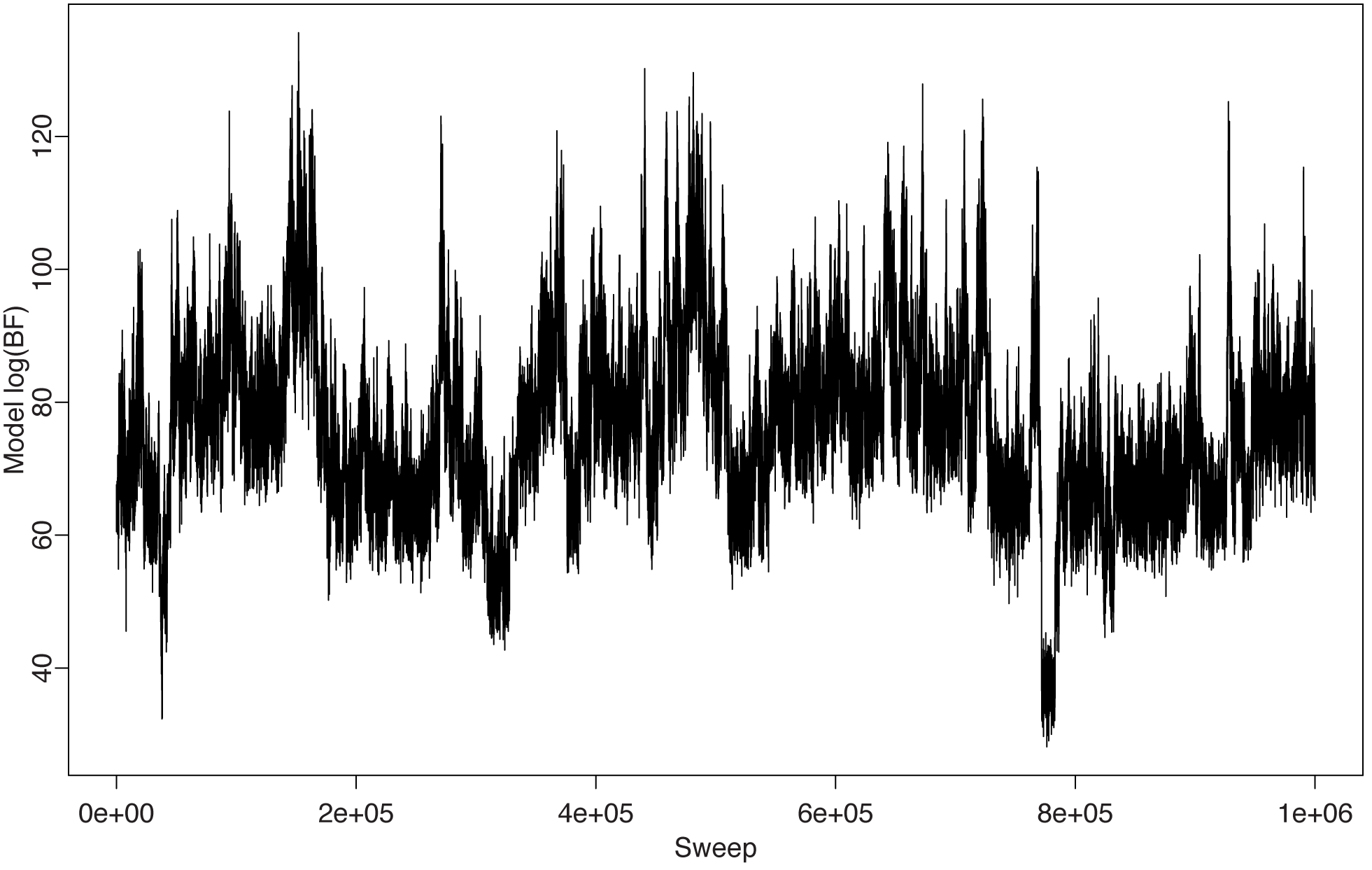

Supplement: Figure S6 — Trace plot of piMASS “Model log10(BF)” in the single trait TG analysis. Values of log10(BF) are recorded every ten iterations. (TIF) [file pgen.1003657.s006.tif]

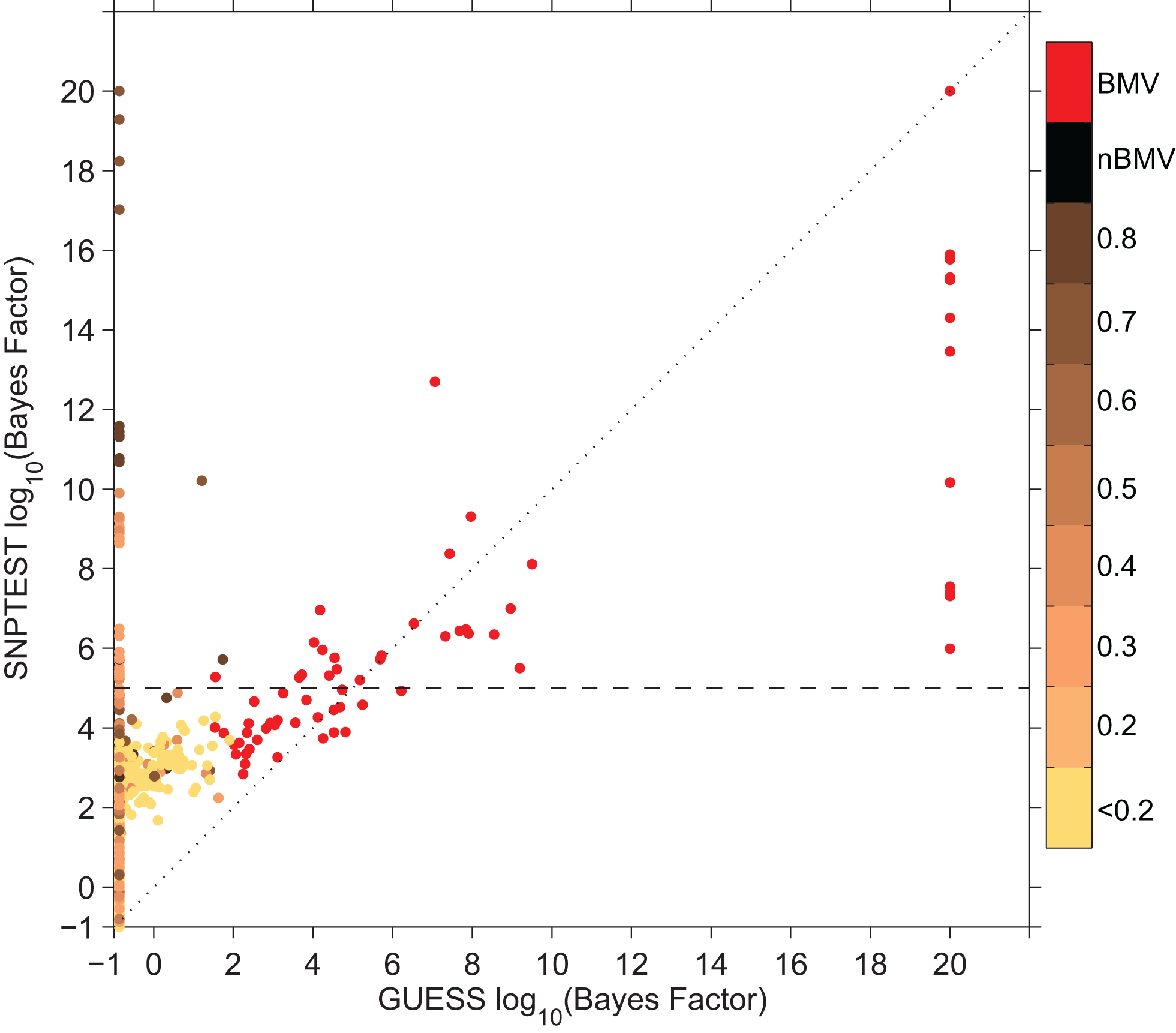

Supplement: Figure S7 — Comparison of the marginal phenotype-SNP association provided by GUESS and SNPTEST for all multiple traits of two trees. (To increase readability, the log10(BFs) are truncated at 20). Scatterplot of log10(BF) GUESS vs SNPTEST obtained superimposing the scatterplot of each multiple trait group. A horizontal dashed line indicates the level of log10(BF) that provides strong evidence of a phenotype-SNP association (log10(BF)>5). Red and black dots highlight significant SNPs found by GUESS while non-significant SNPs are colour coded according to the level of pairwise Pearson correlation with the closest significant GUESS SNP (see the colour bar for the correlation scale). (TIF) [file pgen.1003657.s007.tif]

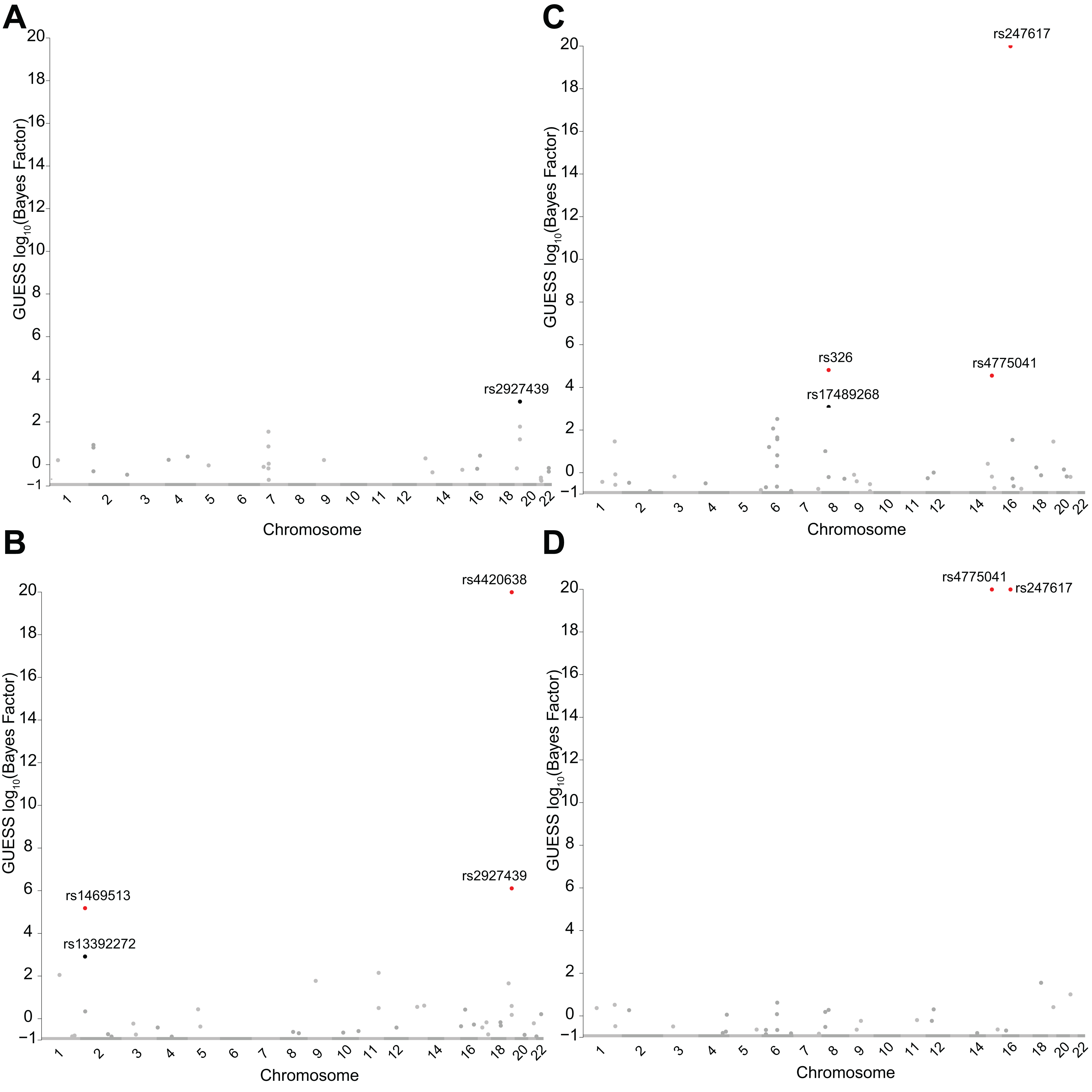

Supplement: Figure S8 — Genome-wide log10(BF) obtained from GUESS for single trait analysis. (A) LDL, (B) APOB (first tree), (C) HDL and (D) APOA1 (second tree). Significant SNPs found associated at a 5% FDR are depicted by black dots (with the SNP's name) whereas significant SNPs that are also in the top Best Model Visited are represented by red dots (with the SNP's name) (the log10(BF) is truncated at 20). (TIF) [file pgen.1003657.s008.tif]

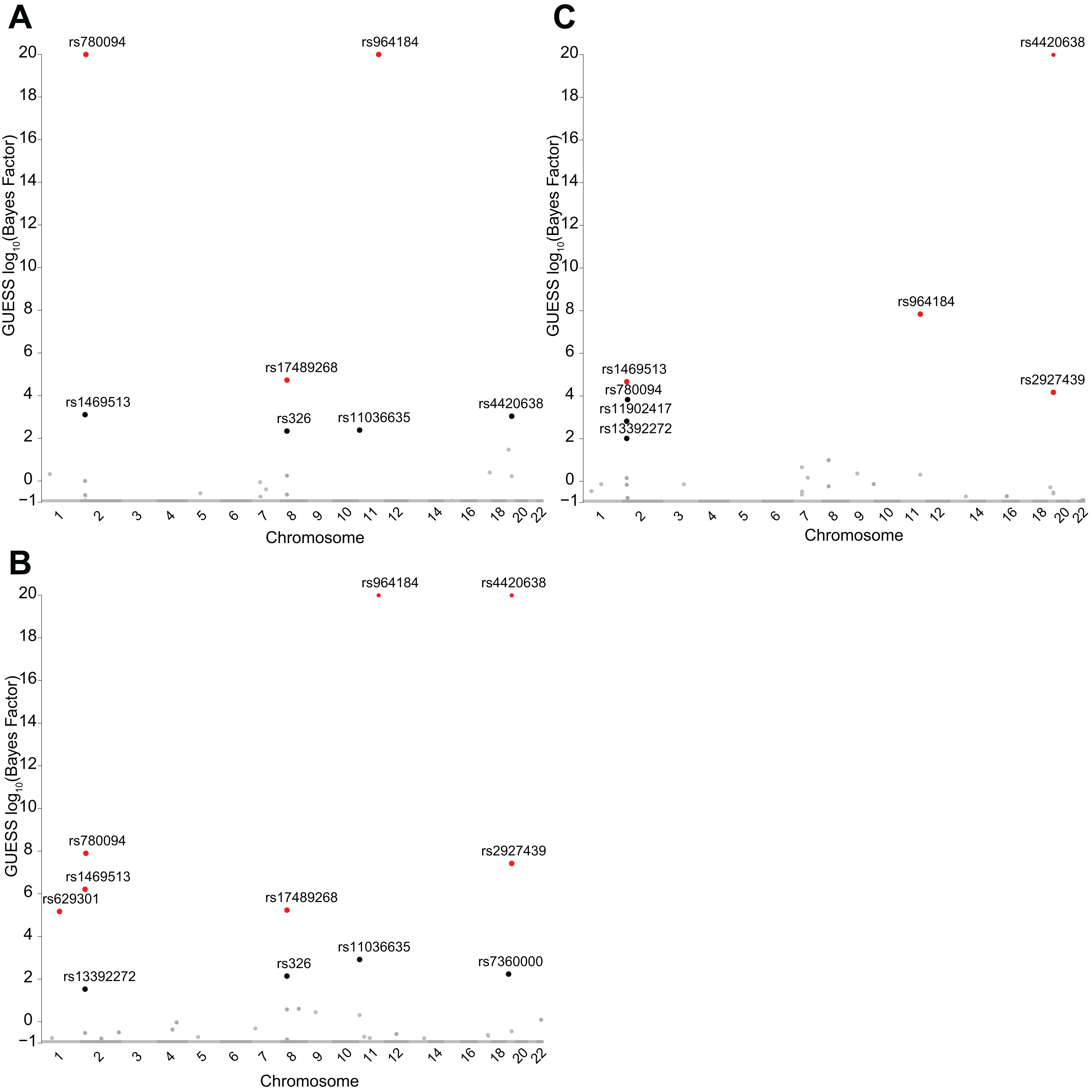

Supplement: Figure S9 — Genome-wide log10(BF) obtained from GUESS in the first tree centred in the LDL pathway. (A) TG-LDL, (B) TG-APOB and (C) LDL-APOB. Significant SNPs found associated at a 5% FDR are depicted by black dots (with the SNP's name) whereas significant SNPs that are also in the top Best Model Visited are represented by red dots (with the SNP's name) (the log10(BF) is truncated at 20). (TIF) [file pgen.1003657.s009.tif]

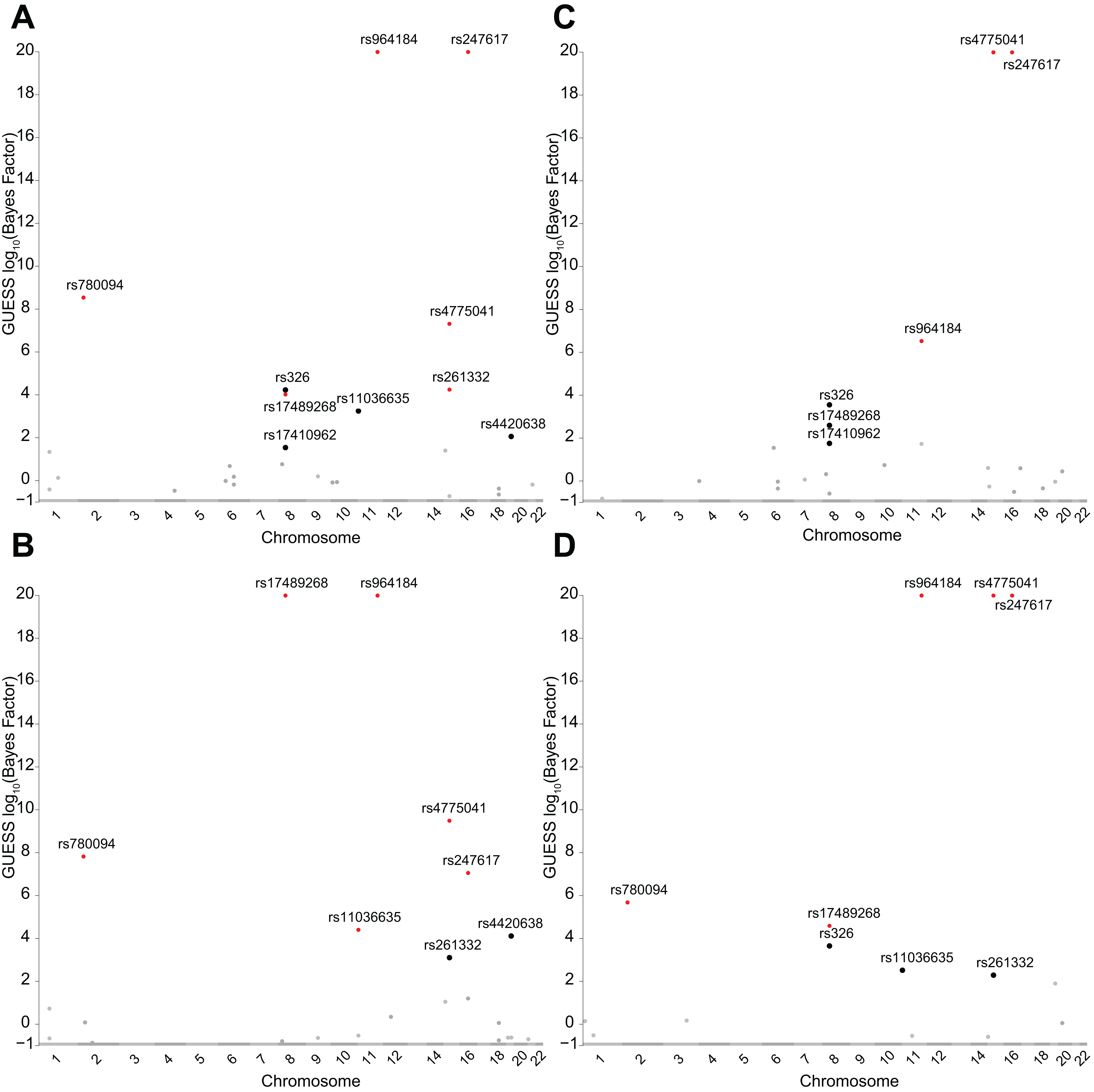

Supplement: Figure S10 — Genome-wide log10(BF) obtained from GUESS in the second tree centred in the HDL pathway. (A) TG-HDL, (B) TG-APOA1, (C) HDL-APOA1 and (D) TG-HDL-APOA1. Significant SNPs found associated at 5% FDR are depicted by black dots (with the SNP's name) whereas significant SNPs that are also in the top Best Model Visited are represented by red dots (with the SNP's name) (the log10(BF) is truncated at 20). (TIF) [file pgen.1003657.s010.tif]

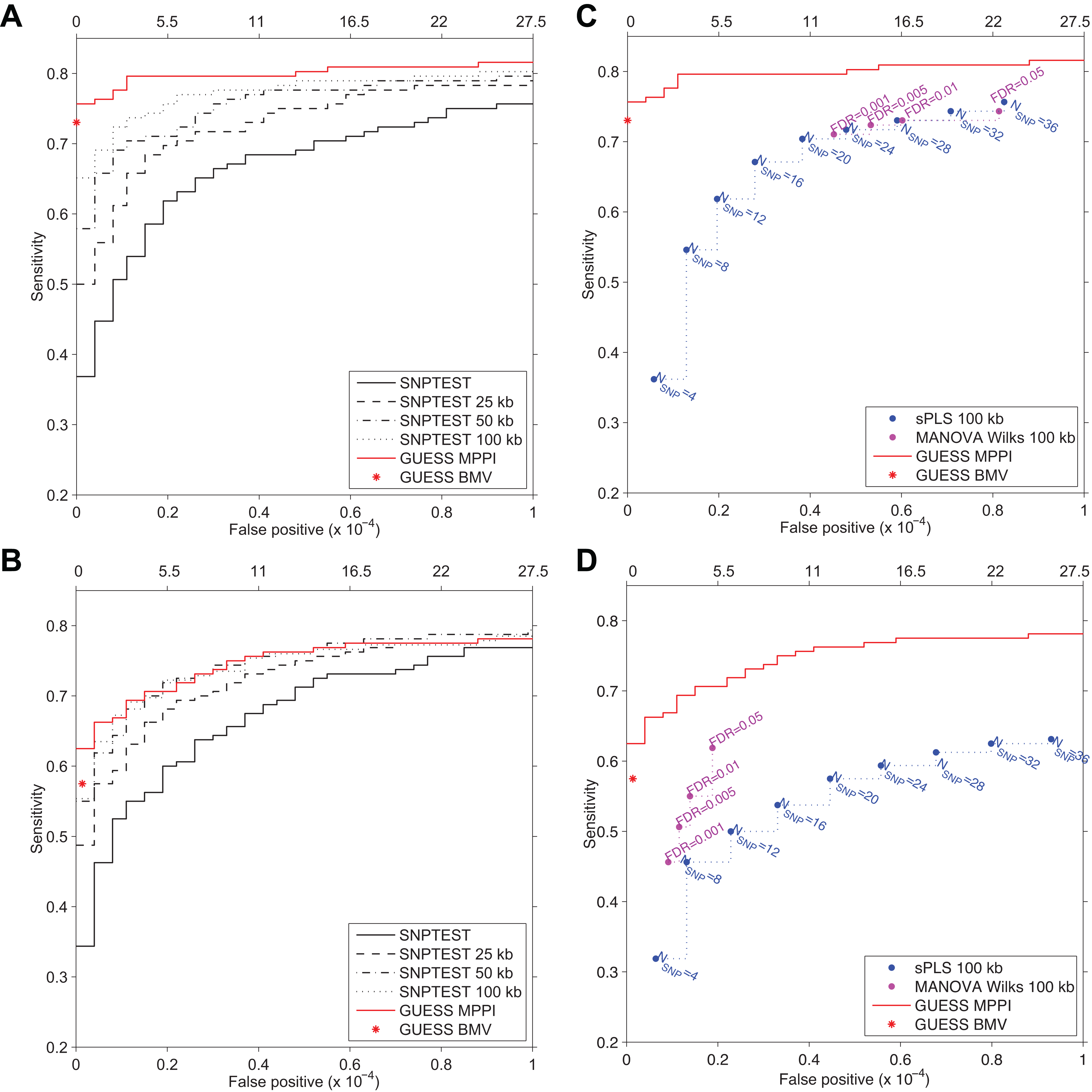

Supplement: Figure S11 — Receiver Operating Characteristic (ROC) curves comparison. ROC curves of SNPTEST (black), SPLS (blue), MANOVA (purple), and GUESS (red) for the first (A–C) and second (B–D) multiple-trait simulated datasets when the definition of positive associations is relaxed, i.e. considering a single association in an interval centred at each top hit and spanning 25 kb, 50 kb and 100 kb on both sides. For GUESS, separate ROC curves are obtained using the top Best Model Visited (red star) and the Marginal Posterior Probability of Inclusion (solid red line). For SNPTEST, the ROC curve is calculated using the log10(BF). The number of SNPs retained by SPLS under different levels of penalization is indicated. For MANOVA Wilks, the ROC curve is derived using SNPs declared significant over a range of FDR levels. The number of false positives (x-axis) is indicated at the top of the figure while the proportion of false positives is presented at the bottom. Given the large number of predictors (273,294), false positives are truncated at 10−4 at which level a large number already occurs (27.5). (TIF) [file pgen.1003657.s011.tif]

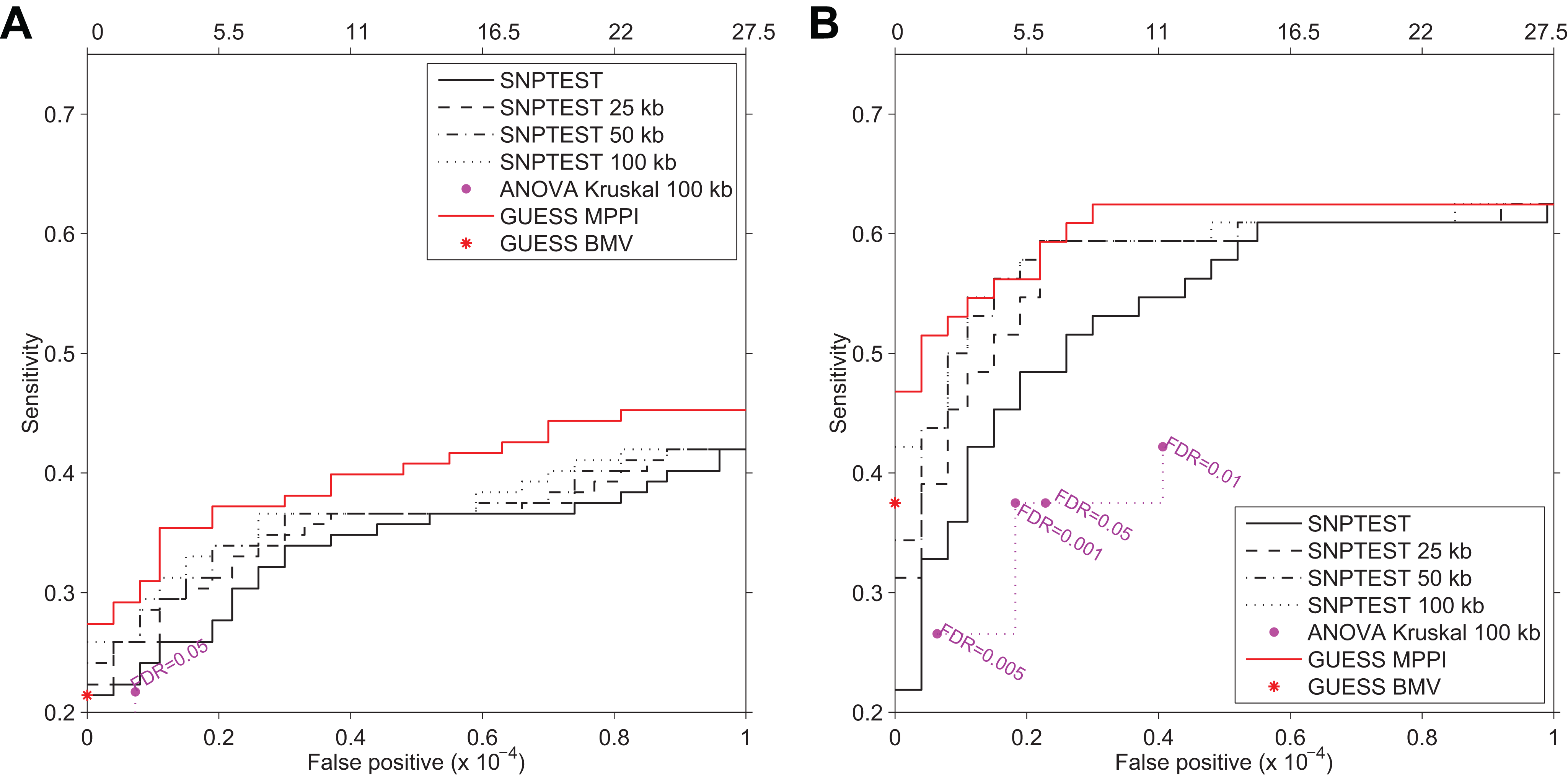

Supplement: Figure S12 — Receiver Operating Characteristic (ROC) curves comparison. ROC curves of SNPTEST (black), ANOVA (purple) and GUESS (red) for the first (A) and second (B) single-trait simulated datasets when the definition of positive associations is relaxed, i.e. considering a single association in an interval centred at each top hit and spanning 25 kb, 50 kb and 100 kb on both sides. For GUESS, separate ROC curves were obtained using the top Best Model Visited (red star) and the Marginal Posterior Probability of Inclusion (MPPI) (solid red line). For SNPTEST, the ROC curve is calculated using the log10(BF). For ANOVA Kruskal, the ROC curve is derived using SNPs declared significant over a range of FDR levels. The number of false positives (x-axis) is indicated at the top of the figure while the proportion of false positives is presented at the bottom. Given the large number of predictors (273,294), false positives are truncated at 10−4 at which level a large number already occurs (27.5). (TIF) [file pgen.1003657.s012.tif]

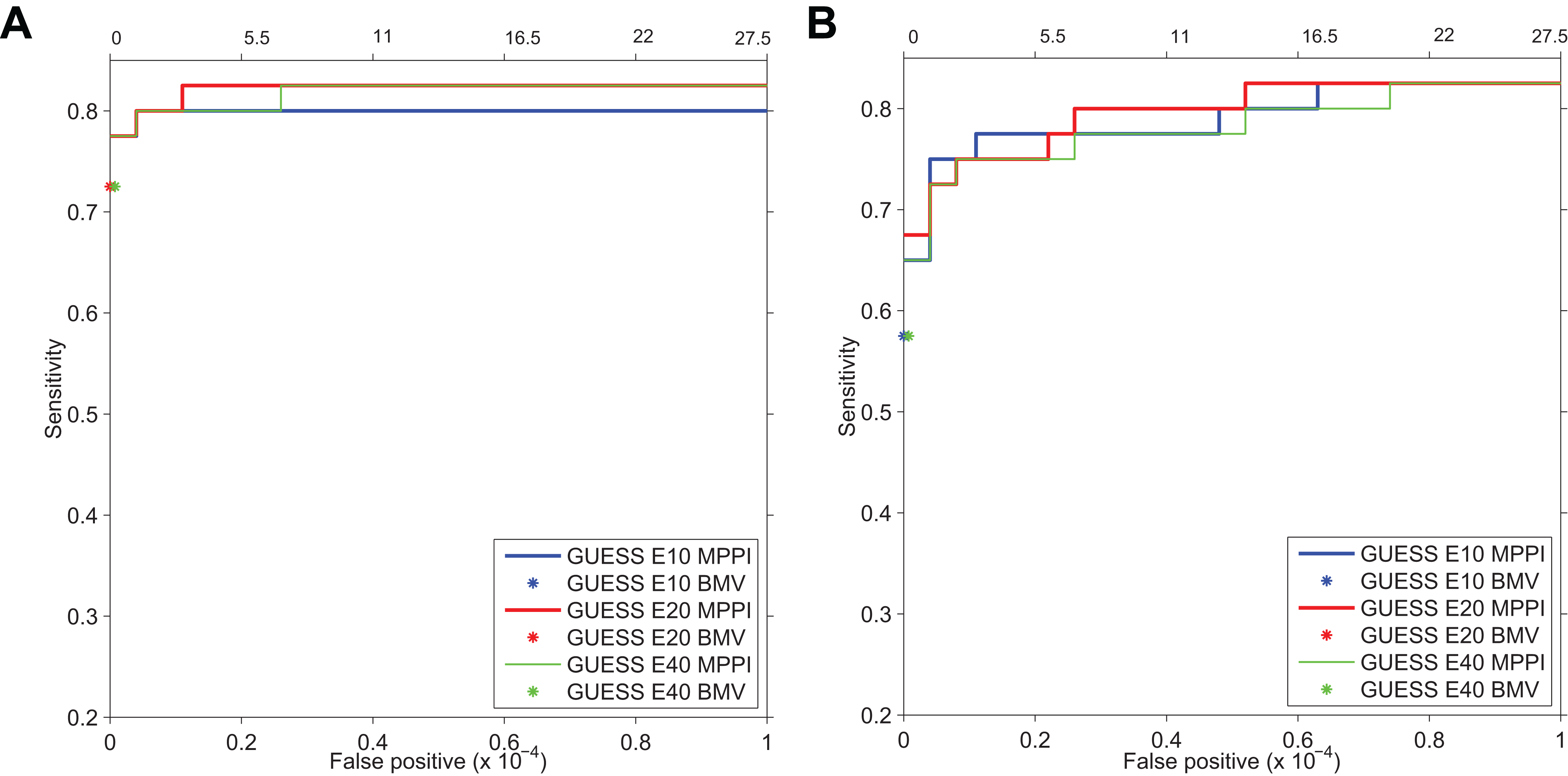

Supplement: Figure S13 — Receiver Operating Characteristic (ROC) curves of GUESS under different parameterization. ROC curve of the a priori expected model size, i.e E = 10 (blue), E = 20 (red) and E = 40 (green) for five replicates of the first (A) and second (B) multi-trait simulated dataset are depicted. Separate ROC curves were obtained using the top Best Model Visited (star) and the Marginal Posterior Probability of Inclusion (solid line). Given the large number of predictors (273,294), false positives are truncated at 10−4 at which level a large number already occurs (27.5). (TIF) [file pgen.1003657.s013.tif]
