# Supplementary material for: GUESS-ing Polygenic Associations with Multiple Phenotypes Using a GPU-Based Evolutionary Stochastic Search Algorithm
Source: PLoS Genet. 2013 Aug 8;9(8):e1003657. doi: 10.1371/journal.pgen.1003657 (PMC3738451; doi:10.1371/journal.pgen.1003657)
Supplement: Table S1 — Post-processed output obtained from GUESS for all the elements of the two trees (green and blue colour coded) and TG-HDL-LDL. Horizontal lines separating groups of traits with the same cardinality (singleton, pairs and triplets). Model Posterior Probability (MPP) of the top Best Model Visited (BMV) and the cumulative MPP of the five top BMV are indicated in the first two columns of the table, respectively. The unique set of significant SNPs (FDR<0.05) which predict a group of phenotypes is indicated on the top of the table as well as the associated locus. Based on Ensembl R66 annotation, each locus is classified as: (1) intronic, (2) 3′UTR, (3) downstream, (4) previously associated and (5) a tagSNP of a previously associated SNP. In the centre of the table log10(RBF), i.e. rescaled marginal phenotype-SNP association, are included with significant SNPs depicted in black and significant SNPs that are also in the top BMV indicated in red (the log10(RBF) is truncated at 20). The Ratio of Bayes Factors (RBF) is a rescaled measure of SNP-trait(s) association and it is defined as the ratio between the BF to test the SNP-trait(s) association hypothesis and the “baseline” BF level obtained through permutations. (PDF) [file pgen.1003657.s014.pdf]

Tree	Trait	Top BMV MPP	Top 5 BMV Cum. MPP	*SORT1*[4] / *CELSR2*[4] / *PSRC1*[2,3,4,5] rs629301 Chr. 1 109,818,306	*APOB*[4,5] rs11902417 Chr. 2 21,198,900	*APOB*[5] rs13392272 Chr. 2 21,217,490	*APOB*[1,4] rs1469513 Chr. 2 21,259,562	*GCKR1*[1,4] rs780094 Chr. 2 27,741,237	*LPL*[1,4,5] / *SLC18A1*[4] rs326 Chr. 8 19,819,439	*LPL*[4] rs17410962 Chr. 8 19,848,080	*LPL*[4,5] rs17489268 Chr. 8 19,852,045	*HBG2*[1] / *HBE1*[1] rs11036635 Chr. 11 5,308,896	*APOA5*[4] / *A4*[4] /*C3*[4] /*A1*[4] *ZNF259*[2,3] rs964184 Chr. 11 116,648,917	*LIPC*[4] *ALDH1A2*[1] rs4775041 Chr. 15 58,674,695	*LIPC*[1,3,4] rs261332 Chr. 15 58,727,325	*CEPT*[5] rs247617 Chr. 16 56,990,716	*MEF2B*[1,5] rs7360000 Chr. 19 19,266,848	rs2927439 Chr. 19 45,242,740	*APOE*[4] /*C1*[3,4] / *C2*[4] /*C4*[4] rs4420638 Chr. 19 45,422,946
TREE I	TG	0.259	0.643					5.409	0.001		2.353	1.868	17.660						
	LDL	0.347	0.454															0.001	
	APOB	0.382	0.573			0.001	2.268											3.195	17.349
	TG-LDL	0.257	0.582				0.766	17.924	0.001		2.388	0.044	17.924						0.697
	TG-APOB	0.264	0.525	3.638		0.001	4.679	6.366	1.201		3.704	1.388	18.731				0.704	5.890	18.731
	LDL-APOB	0.164	0.536		0.807	0.001	2.659	1.821					5.828					2.160	18.253
	TG-LDL-APOB	0.314	0.692	3.397			2.206	6.869	0.001		2.201		17.950					5.643	17.950
TREE II	TG	0.259	0.643					5.409	0.001		2.353	1.868	17.660						
	HDL	0.214	0.494						1.734		0.000			1.472		17.375			
	APOA1	0.583	0.651											12.136		12.136			
	TG-HDL	0.140	0.550					6.997	2.689	0.001	2.478	1.706	18.722	5.772	2.704	18.722			0.519
	TG-APOA1	0.185	0.673					6.366			17.158	1.297	17.158	6.386	0.001	3.948			1.009
	HDL-APOA1	0.402	0.763						1.799	0.001	0.840		4.771	18.510		18.510			
	TG-HDL-APOA1	0.295	0.678					3.781	1.752		2.690	0.618	18.365	18.365	0.389	18.365			
	**TG-HDL-LDL**	0.314	0.757				0.001	17.868	1.734		2.142		17.868	5.281	2.414	17.868			6.560
