# Supplementary material for: GUESS-ing Polygenic Associations with Multiple Phenotypes Using a GPU-Based Evolutionary Stochastic Search Algorithm
Source: PLoS Genet. 2013 Aug 8;9(8):e1003657. doi: 10.1371/journal.pgen.1003657 (PMC3738451; doi:10.1371/journal.pgen.1003657)
Supplement: Table S2 — Comparison of prior setup between GUESS, SNPTEST and piMASS. In SNPTEST the hyper-priors on π and on the variance of the regression coefficients are not specified. piMASS differs from GUESS by a different specification of the priors on the regression coefficients and on their variance. (PDF) [file pgen.1003657.s015.pdf]

	GUESS	SNPTEST	piMASS
**Sparsity prior**	Binomial prior with hyper-prior π	Bernoulli prior Probability π fixed at 10^{-4}	Bernoulli prior with hyper-prior π
**Hyper-prior on π**	Beta prior User can specify hyper-parameters	No	Log-Uniform prior User can specify hyper-parameters
**Prior on error variance**	InvGamma/ InvWishart prior. Conjugate with the effects variance	InvGamma/ InvWishart prior. Conjugate with the effects variance	InvGamma prior. Conjugate with the effects variance
**Hyper-parameters on prior error variance**	Diffuse prior specified. For InvWishart automatic empirical Bayes estimates	Diffuse prior specified	Diffuse prior specified
**Prior on regression coefficients**	Normal/Matrix normal *g*-prior	Normal/Matrix normal independent prior	Normal independent prior
**Prior on effects variance**	InvGamma/ InvWishart prior	InvGamma/ InvWishart prior	InvGamma prior
**Hyper-prior on variance of regression coefficients**	InvGamma prior (with no existing moments and heavy tails)	No	Special form (heavy tails)
