# Supplementary material for: GUESS-ing Polygenic Associations with Multiple Phenotypes Using a GPU-Based Evolutionary Stochastic Search Algorithm
Source: PLoS Genet. 2013 Aug 8;9(8):e1003657. doi: 10.1371/journal.pgen.1003657 (PMC3738451; doi:10.1371/journal.pgen.1003657)
Supplement: Table S3 — Comparison of the MCMC efficiency between GUESS and piMASS. GUESS was run for 100,000 sweeps with 10,000 as burn-in and with 3 chains. piMASS was run for 1.1×10^7^ iterations with 106 as burn-in. GUESS analysis was performed on an HPC cluster computer with a 2.8 GHz Dual-Core Xeon processor and a NVidia Tesla C1060 GPU with 8 Gb of RAM, while piMASS was run on a 3 GHz computer with a 1024 KB cache size Dual-Core AMD Opteron processor and 16 Gb of RAM. “Computational time” is reported in hours (rounded to the nearest integer). “Number of models evaluated” includes the burn-in phase, while “Number of unique model visited” and “Number of model visited before (visiting) top Best Model Visited” are calculated after the burn-in phase. “Average model size” is the average dimension (standard deviation in brackets) of the model recorded in GUESS (from the non-heated chain) and piMASS (every 10 iterations). For piMASS the number of models evaluated corresponds to the number of iterations and is roughly equal to a third of the models evaluated by GUESS. (PDF) [file pgen.1003657.s016.pdf]

Groups of traits	GUESS					piMASS			
	Comp. time hours	# models evaluated	# unique models visited	# models visited before top BMV	Average (and std) model size	Comp. time hours	# unique models visited	# models visited before top BMV	Average (and std) model size
**APOA1**	255	33,421,374	47,285	363	3.86 (1.51)	632	519,662	775,127	12.43 (12.47)
**APOB**	283	36,174,089	53,492	5,746	6.22 (2.31)	635	619,055	395,125	21.54 (15.04)
**HDL**	261	34,054,212	49,188	2,957	3.89 (1.94)	643	598,881	250,669	20.48 (14.44)
**LDL**	194	34,316,607	46,587	1,915	2.62 (1.84)	625	682,042	145,260	19.03 (16.92)
**TG**	265	34,718,263	52,205	1,771	5.74 (1.81)	675	624,890	151,931	10.41 (7.84)
**HDL-APOA1**	201	34,146,989	18,496	86	4.17 (1.02)	-	-	-	-
**LDL-APOB**	268	34,213,582	25,778	611,784	4.41 (1.32)	-	-	-	-
**TG-APOA1**	212	34,504,619	27,395	9,440	6.96 (1.24)	-	-	-	-
**TG-APOB**	208	34,209,888	32,021	6,949	7.63 (1.35)	-	-	-	-
**TG-HDL**	282	34,284,477	26,260	14,876	6.77 (1.32)	-	-	-	-
**TG-LDL**	265	33,559,719	22,067	298,405	4.07 (1.33)	-	-	-	-
**TG-HDL-APOA1**	233	33,723,713	8,237	309	5.38 (0.90)	-	-	-	--
**TG-HDL-LDL**	196	34,054,533	9,729	207	7.02 (0.89)	-	-	-	-
**TG-LDL-APOB**	201	33,376,005	9,636	290	6.21 (1.09)	-	-	-	-
