# Supplementary material for: GUESS-ing Polygenic Associations with Multiple Phenotypes Using a GPU-Based Evolutionary Stochastic Search Algorithm
Source: PLoS Genet. 2013 Aug 8;9(8):e1003657. doi: 10.1371/journal.pgen.1003657 (PMC3738451; doi:10.1371/journal.pgen.1003657)
Supplement: Table S4 — Output obtained from SNPTEST for all elements of the two trees (green and blue colour coded) and TG-HDL-LDL. Horizontal lines separating groups of traits with the same cardinality (singleton, pairs and triplets). The unique set of significant SNPs (FDR<0.05) found by GUESS which predict a group of phenotypes is indicated on the top of the table as well as the associated locus. Based on Ensembl R66 annotation, each locus is classified as: (1) intronic, (2) 3′UTR, (3) downstream, (4) previously associated and (5) tagSNP of previously associated SNP. In the centre of the table the SNPTEST log10(Bayes Factor) for significant SNPs found associated by GUESS is included (the log10(BF) is truncated at 20). (PDF) [file pgen.1003657.s017.pdf]

Tree	Trait	*SORT1*[4] / *CELSR2*[4] / *PSRC1*[2,3,4,5] rs629301 Chr. 1 109,818,306	*APOB*[4,5] rs11902417 Chr. 2 21,198,900	*APOB*[5] rs13392272 Chr. 2 21,217,490	*APOB*[1,4] rs1469513 Chr. 2 21,259,562	*GCKR1*[1,4] rs780094 Chr. 2 27,741,237	*LPL*[1,4,5] / *SLC18A1*[4] rs326 Chr. 8 19,819,439	*LPL*[4] rs17410962 Chr. 8 19,848,080	*LPL*[4,5] rs17489268 Chr. 8 19,852,045	*HBG2*[1] / *HBE1*[1] rs11036635 Chr. 11 5,308,896	*APOA5*[4] / *A4*[4] /*C3*[4] /*A1*[4] *ZNF259*[2,3] rs964184 Chr. 11 116,648,917	*LIPC*[4] *ALDH1A2*[1] rs4775041 Chr. 15 58,674,695	*LIPC*[1,3,4] rs261332 Chr. 15 58,727,325	*CEPT*[5] rs247617 Chr. 16 56,990,716	*MEF2B*[1,5] rs7360000 Chr. 19 19,266,848	rs2927439 Chr. 19 45,242,740	*APOE*[4] /*C1*[3,4] / *C2*[4] /*C4*[4] rs4420638 Chr. 19 45,422,946
**TREE I**	**TG**					6.654	4.231		5.018	4.844	15.891						
	**LDL**															4.259	
	**APOB**			4.059	4.992											7.370	10.740
	**TG-LDL**				4.191	7.312	3.876		4.958	4.113	15.255						4.075
	**TG-APOB**	5.202		4.010	4.931	6.370	3.625		4.580	4.123	15.774				2.841	8.373	10.169
	**LDL-APOB**		3.988	4.518	4.518	4.698			6.622		6.622					6.956	13.458
	**TG-LDL-APOB**	5.816			3.884	5.501	3.348		4.449		15.858					9.309	14.302
**TREE II**	**TG**					6.654	4.231		5.018	4.844	15.891						
	**HDL**						4.689		4.140			4.971		20.000			
	**APOA1**											8.043		13.435			
	**TG-HDL**					6.345	5.957	5.274	6.149	4.876	15.785	6.301	3.734	20.000			3.332
	**TG-APOA1**					6.370			5.988	5.315	6.469	8.110	3.258	12.696			4.267
	**HDL-APOA1**						4.128	3.865	3.699		3.388	7.548		20.000			
	**TG-HDL-APOA1**					5.725	5.267		5.471	4.660	15.317	7.401	3.096	20.000			
	**TG-HDL-LDL**				3.464	7.397	5.338		5.759		15.205	6.437	3.893	20.000			6.998
