# Supplementary material for: GUESS-ing Polygenic Associations with Multiple Phenotypes Using a GPU-Based Evolutionary Stochastic Search Algorithm
Source: PLoS Genet. 2013 Aug 8;9(8):e1003657. doi: 10.1371/journal.pgen.1003657 (PMC3738451; doi:10.1371/journal.pgen.1003657)
Supplement: Table S5 — Results of the empirical FDR procedure. For each element of the two trees centred on the LDL and HDL pathways and TG-HDL-LDL, we report the sample size of the null distribution used in the empirical FDR procedure that we obtained combining the Marginal Posterior Probability of Inclusion (MPPI) for all the artificial groups of phenotypes with the same dimension (each element of the trees was permuted 3 times and the MPPI of all artificial groups of traits with the same dimension, i.e. 5 singleton (+), 6 pairs (++) and 3 triplets (+++), were used to calculate the empirical FDR), the MPPI threshold at an FDR of 5% and the number of significant SNPs associated with each group of phenotypes. (PDF) [file pgen.1003657.s018.pdf]

Groups of traits	Tree centred on pathway	Sample size null distribution (# artificial data sets)	MPPI threshold at 5% FDR	$\log_{10}$(BF) threshold at 5% FDR	Significant SNPs called at 5% FDR
**APOA1[+]**	HDL	4,099,410 (3×5)	0.1077	3.192	2
**APOB[+]**	LDL	4,099,410 (3×5)	0.0577	2.923	4
**HDL[+]**	HDL	4,099,410 (3×5)	0.0822	3.088	4
**LDL[+]**	LDL	4,099,410 (3×5)	0.0632	2.965	1
**TG[+]**	HDL/LDL	4,099,410 (3×5)	0.0291	2.612	5
**HDL-APOA1[++]**	HDL	4,919,292 (3×6)	0.0042	1.762	6
**LDL-APOB[++]**	LDL	4,919,292 (3×6)	0.0076	2.019	7
**TG-APOA1[++]**	HDL	4,919,292 (3×6)	0.0868	3.114	8
**TG-APOB[++]**	LDL	4,919,292 (3×6)	0.0025	1.541	11
**TG-HDL[++]**	HDL	4,919,292 (3×6)	0.0026	1.550	10
**TG-LDL[++]**	LDL	4,919,292 (3×6)	0.0160	2.348	7
**TG-HDL-APOA1[+++]**	HDL	2,459,646 (3×3)	0.0059	1.960	9
**TG-HDL-LDL[+++]**	-	2,459,646 (3×3)	0.0182	2.404	9
**TG-LDL-APOB[+++]**	LDL	2,459,646 (3×3)	0.0151	2.322	8
