# Supplementary material for: GUESS-ing Polygenic Associations with Multiple Phenotypes Using a GPU-Based Evolutionary Stochastic Search Algorithm
Source: PLoS Genet. 2013 Aug 8;9(8):e1003657. doi: 10.1371/journal.pgen.1003657 (PMC3738451; doi:10.1371/journal.pgen.1003657)
Supplement: Table S6 — Genetic associations for selected phenotypic groups (TG-APOB and TG-LDL-APOB in “Tree I”; TG-HDL in “Tree II” and TH-HDL-LDL) detected by GUESS. Two independent replication datasets were used (a) Copenhagen City Heart Study (CCHS) and (b) Data from an Epidemiological Study on the Insulin Resistance syndrome (DESIR). Each region centred at the identified causal variant in the discovery dataset (rs629301 and rs261333, respectively) and spanning 2 Mb is regressed against the phenotypic groups previously associated with the variant. Genetic markers with the largest significant BF obtained with SNPTEST in each region are reported in each table as well as their position in the genome. SNP rs261332 is not present in the CardioMetabochip. Using 381 Caucasian individuals from the 1000 Genomes project, r2 and D′ are 0.582 and 0.979 between rs261332 and rs8034802 in (A) and 0.838 and 0.982 between rs261332 and rs1077834 in (B), respectively. (PDF) [file pgen.1003657.s019.pdf]

A

		Region around rs629301 Chr. 1 109,818,306	Region around rs261332 Chr. 15 58,727,325
TREE I	TG<br>LDL<br>APOB		
TREE I	TG-LDL<br>TG-APOB<br>LDL-APOB	**rs629301** 109,818,306	
TREE I	TG-LDL-APOB	**rs629301** 109,818,306	
TREE II	TG<br>HDL<br>APOA1		
TREE II	TG-HDL<br>TG-APOA1<br>HDL-APOA1		**rs8034802** 58,724,792
TREE II	TG-HDL-APOA1		
	TG-HDL-LDL		**rs8034802** 58,724,792

B

		Region around rs629301 Chr. 1 109,818,306	Region around rs261332 Chr. 15 58,727,325
TREE I	TG<br>LDL<br>APOB		
TREE I	TG-LDL<br>TG-APOB<br>LDL-APOB	**rs629301** 109,818,306	
TREE I	TG-LDL-APOB	**rs629301** 109,818,306	
TREE II	TG<br>HDL<br>APOA1		
TREE II	TG-HDL<br>TG-APOA1<br>HDL-APOA1		**rs1077834** 58,723,479
TREE II	TG-HDL-APOA1		
	TG-HDL-LDL		**rs1077834** 58,723,479
