# Supplementary material for: GUESS-ing Polygenic Associations with Multiple Phenotypes Using a GPU-Based Evolutionary Stochastic Search Algorithm
Source: PLoS Genet. 2013 Aug 8;9(8):e1003657. doi: 10.1371/journal.pgen.1003657 (PMC3738451; doi:10.1371/journal.pgen.1003657)
Supplement: Table S7 — Strength of the genetic association provided by the RBF between genetic variants identified in Table S6 and the branches of the two trees and TG-HDL-LDL. Two independent replication datasets were used (a) Copenhagen City Heart Study (CCHS) and (b) Data from an Epidemiological Study on the Insulin Resistance syndrome (DESIR) (computations of RBF based on the SNPTEST BF). Combinations of phenotypic groups and genetic markers previously found to be most associated in the discovery data set Gutenberg Health Study (GHS) are highlighted in bold. A dashed line indicates that the genetic association is not significant at a 5% FDR in the selected 2 Mb region. (PDF) [file pgen.1003657.s020.pdf]

A

		rs629301<br>Chr. 1<br>109,818,306	rs8034802<br>Chr. 15<br>58,724,792
TREE I	TG	-	
	LDL	7.786	
	APOB	9.272	
	TG-LDL	6.653	
	TG-APOB	**9.892**	
	LDL-APOB	8.152	
	TG-LDL-APOB	**9.510**	
TREE II	TG		-
	HDL		12.968
	APOA1		14.208
	TG-HDL		**17.490**
	TG-APOA1		12.018
	HDL-APOA1		12.386
	TG-HDL-APOA1		10.641
	**TG-HDL-LDL**		**17.716**

B

		rs629301<br>Chr. 1<br>109,818,306	rs1077834<br>Chr. 15<br>58,723,479
TREE I	TG	-	
	LDL	5.660	
	APOB	5.470	
	TG-LDL	5.141	
	TG-APOB	**6.761**	
	LDL-APOB	6.295	
	TG-LDL-APOB	**5.855**	
TREE II	TG		-
	HDL		13.213
	APOA1		12.961
	TG-HDL		**22.192**
	TG-APOA1		16.977
	HDL-APOA1		13.183
	TG-HDL-APOA1		19.947
	**TG-HDL-LDL**		**22.130**
