# Supplementary material for: GUESS-ing Polygenic Associations with Multiple Phenotypes Using a GPU-Based Evolutionary Stochastic Search Algorithm
Source: PLoS Genet. 2013 Aug 8;9(8):e1003657. doi: 10.1371/journal.pgen.1003657 (PMC3738451; doi:10.1371/journal.pgen.1003657)
Supplement: Table S8 — Post-processed output obtained from GUESS with different prior specification. For selected elements of the two trees (green and blue colour coded) and TG-HDL-LDL GUESS was run using a conjugate hierarchical independent prior for the genetic effects with a diffuse exponential hyper-prior for the variance of the regression coefficients. Model Posterior Probability (MPP) of the top Best Model Visited (BMV) and the cumulative MPP of the five top BMV are indicated in the first two columns of the table, respectively. The previously identified unique sets of significant SNPs (FDR<0.05) which predict a group of phenotypes is indicated on the top of the table as well as the associated locus. Based on Ensembl R66 annotation, each locus previously identified is classified as: (1) intronic, (2) 3′UTR, (3) downstream, (4) previously associated and (5) a tagSNP of a previously associated SNP. SNP-trait(s) association identified in the BMV by GUESS with the new prior specification are presented in the centre of the table. The BMV for the selected elements of the two trees are as depicted in Table S1. (PDF) [file pgen.1003657.s021.pdf]

		Top BMV MPP	Top 5 BMV Cum. MPP	*SORT1*[4] / *CELSR2*[4] / *PSRC1*[2,3,4,5] rs629301 Chr. 1 109,818,306	*APOB*[4,5] rs11902417 Chr. 2 21,198,900	*APOB*[5] rs13392272 Chr. 2 21,217,490	*APOB*[1,4] rs1469513 Chr. 2 21,259,562	*GCKR1*[1,4] rs780094 Chr. 2 27,741,237	*LPL*[1,4,5] / *SLC18A1*[4] rs326 Chr. 8 19,819,439	*LPL*[4] rs17410962 Chr. 8 19,848,080	*LPL*[4,5] rs17489268 Chr. 8 19,852,045	*HBG2*[1] / *HBE1*[1] rs11036635 Chr. 11 5,308,896	*APOA5*[4] / *A4*[4] /*C3*[4] /*A1*[illegible] *ZNF259*[2,3] rs964184 Chr. 11 116,648,917	*LIPC*[4] *ALDH1A2*[1] rs4775041 Chr. 15 58,674,695	*LIPC*[1,3,4] rs261332 Chr. 15 58,727,325	*CEPT*[5] rs247617 Chr. 16 56,990,716	*MEF2B*[1,5] rs7360000 Chr. 19 19,266,848	rs2927439 Chr. 19 45,242,740	*APOE*[4] /*C1*[3,4] / *C2*[4] /*C4*[4] rs4420638 Chr. 19 45,422,946
TREE I	**TG-LDL-APOB**	0.357	0.675	✔			✔	✔			✔		✔					✔	✔
TREE II	**TG-HDL-APOA1**	0.295	0.678					✔			✔		✔	✔		✔			
	**TG-HDL-LDL**	0.337	0.842					✔			✔		✔	✔	✔	✔			✔
